# Age and Sex Differences in Heart Failure With Preserved Ejection Fraction

**DOI:** 10.3389/fragi.2022.811436

**Published:** 2022-02-15

**Authors:** Kamil Aleksander Kobak, Weronika Zarzycka, Ying Ann Chiao

**Affiliations:** Aging and Metabolism Research Program, Oklahoma Medical Research Foundation, Oklahoma City, OK, United States

**Keywords:** sex differences, animal models, heart failure with preserved ejection fraction (HFpEF), diastolic dysfunction, cardiac aging

## Abstract

Heart failure with preserved ejection fraction (HFpEF) is a multi-organ disorder that represents about 50% of total heart failure (HF) cases and is the most common form of HF in the elderly. Because of its increasing prevalence caused by the aging population, high mortality and morbidity, and very limited therapeutic options, HFpEF is considered as one of the greatest unmet medical needs in cardiovascular medicine. Despite its complex pathophysiology, numerous preclinical models have been established in rodents and in large animals to study HFpEF pathophysiology. Although age and sex differences are well described in HFpEF population, there are knowledge gaps in sex- and age-specific differences in established preclinical models. In this review, we summarize various strategies that have been used to develop HFpEF models and discuss the knowledge gaps in sex and age differences in HFpEF.

## Introduction

Heart failure (HF) is a leading cause of death worldwide and its prevalence is estimated to be 1%–2% of the adult population in developed countries, rising to ≥10% among people over 70 years of age (McDonagh et al., 2021). HF is a chronic, progressive condition in which the heart muscle is unable to pump enough blood to meet the body’s need for blood and oxygen. Typical symptoms of HF are exercise intolerance, fatigue and weakness, congestion, dyspnea, and peripheral edema (McDonagh et al., 2021). Diverse etiologies of HF can be associated with reduced, preserved, or mid-range left ventricular ejection fraction (LVEF). HF with reduced ejection fraction (HFrEF; LVEF <40%) is typically associated with myocardial damage–related loss of cardiomyocytes and results in the inability of the left ventricle to contract properly (Simmonds et al., 2020). Pathophysiology of HF with preserved ejection fraction (HFpEF; LVEF >50%) is more complex and is not as well defined. Although, historically, HFpEF was called diastolic HF, it is now recognized that HFpEF is a multi-organ disorder because this systemic syndrome involves multiple pathophysiological abnormalities above and beyond LV diastolic dysfunction. Multiple derangements in metabolism and cardiac, pulmonary, renal, skeletal, and immune systems coordinately contribute to the development of HFpEF symptoms and outcomes (Mishra and Kass, 2021). In particular, the myocardium of patient with HFpEF is characterized by structural remodeling and cellular abnormalities such as cardiomyocyte hypertrophy, fibrosis, and inflammation that eventually lead to diastolic dysfunction of left ventricle (Mishra and Kass, 2021). HF with mid-range ejection fraction (HFmrEF; LVEF 40%–50%) is an intermediate state between HFrEF and HFpEF (Kapoor et al., 2016; Nadruz et al., 2016). Differences in the pathological development of HFrEF and HFpEF have been summarized and discussed in recent review articles (Mentz et al., 2014; Simmonds et al., 2020).

HFpEF consists of about 50% of total HF cases (Dunlay et al., 2017), and its prevalence relative to HFrEF is predicted to increase at a rate of 1% per year, indicating that HFpEF is becoming the most common type of HF (Steinberg et al., 2012; Oktay et al., 2013). HFpEF is associated with high morbidity and mortality with a 2-year risk of hospitalization or all-cause death of 35% and 2-year mortality of 14%, which is similar or slightly below as in HFrEF (Lam et al., 2018). Epidemiological data showed that the prevalence of HFpEF is very low in people aged 55 or younger but increases sharply with age thereafter and reaches >8% in women over 80 years of age (Dunlay et al., 2017; Kitzman et al., 2017). The Baltimore Longitudinal Study on Aging has showed that aging in healthy men and women is associated with LV hypertrophy, declined diastolic function, preserved systolic function at rest, and reduced cardiac reserve (Lakatta and Levy, 2003). These age-related changes in cardiac function overlap with the cardiac changes observed in patients with HFpEF (Mishra and Kass, 2021). Moreover, aging contributes to molecular and cellular alterations in the heart (Loffredo et al., 2014; Chiao and Rabinovitch, 2015), many of which have been also implicated in HFpEF (Samson et al., 2016; Mishra and Kass, 2021). These observations highlight the important role of aging in HFpEF pathogenesis. Three independent HFpEF trials have shown that the elderly patient group was predominantly women with a high incidence of comorbidities (Yusuf et al., 2003; Massie et al., 2008; Pitt et al., 2014; Tromp et al., 2019). The higher occurrence of HFpEF in women suggests that sex-dependent mechanisms may be involved in HFpEF pathogenesis (Dunlay et al., 2017). The high prevalence of HFpEF in postmenopausal women also hints that menopause-related estrogen dysregulation and the related comorbidities, such as obesity, diabetes, hypertension, and renal dysfunction, may contribute to HFpEF development (Sabbatini and Kararigas, 2020). Because of the longer life expectancy of women compared to men, there is a higher percentage of women than men in the older age groups. This age distribution of the population may also contribute to the higher percentage of women with HFpEF. A recent study showed that, after adjusting for age, obesity (body mass index), blood pressure, current treatment for hypertension, and previous myocardial infarction, the risk of HFpEF is similar in men and women (Ho et al., 2016). Further investigations are required to determine the sex differences and sex-dependent mechanisms of HFpEF.

Extensive clinical and preclinical studies have resulted in the development of efficient therapies for HFrEF (Vaduganathan et al., 2020). However, these treatments mostly are not effective for HFpEF management, and new evidence-based treatments for HFpEF are needed. On the basis of experimental and clinical data, several cellular and molecular mechanisms such as cardiac hypertrophy, myocardial fibrosis, sarcomere dysfunction, reduced cGMP-PKG signaling, nitrosative-oxidative stress, and mitochondrial and metabolic defects have been proposed to mediate the pathophysiology of HFpEF (Yamamoto et al., 2002; van Heerebeek et al., 2008; Phan et al., 2009; Zile et al., 2011; Chirinos and Zamani, 2016; Schiattarella et al., 2019). These mechanisms of HFpEF have been nicely summarized in a recent review (Mishra and Kass, 2021). A recent study using a mouse model of HFpEF showed that metabolic drugs, including sodium-glucose co-transporter 2 inhibitor (SGLT2i) dapagliflozin, are potential treatments for HFpEF (Withaar et al., 2021a). Interestingly, the EMPEROR-Preserved clinical trial also showed that SGLT2i empagliflozin reduces the risk of cardiovascular death or hospitalization for HF in patients with HFpEF (Anker et al., 2021). The consistent results of these preclinical and clinical studies highlight the potential of preclinical animal studies to identify the mechanisms and therapeutic targets for HFpEF.

HFpEF is recognized to be a multi-systemic disorder with broad phenotypic heterogeneity (Juillière et al., 2018). Prognoses and outcomes of HFpEF are strongly correlated with the presence of multiple chronic comorbidities such as hypertension, obesity, diabetes, and atrial fibrillation (Mentz et al., 2014; Dunlay et al., 2017; Pfeffer et al., 2019). Because of the multimorbidity and heterogeneity of patients with HFpEF (Juillière et al., 2018), preclinical studies should consider the multiorgan complexity of this syndrome. Although numerous animal models have been used to study the pathogenic mechanisms of HFpEF, many HFpEF models do not recapitulate the systemic pathophysiology of patients with HFpEF. In this article, we summarize the available animal models and discuss the knowledge gaps in the age and sex differences in HFpEF (Table 1).

**TABLE 1:** Selected small and large animal preclinical models used to study HFpEF.

Stress factor	Animal model	Age group	Age at endpoint	Sex	Sex differences	Cardiac phenotype				Extracardiac phenotype		Remarks	Ref
						Preserved EF	Diastolic dysfunction	Cardiac hypertrophy	Cardiac fibrosis	Lung congestion	Exercise intolerance		
Aging	Senescence-accelerated mouse (SAM)	Young	24 weeks	F	ND	+	+	+	+	ND	+	-	Gevaert et al. (2017)
	Aged C57BL/6 mouse	Old	24–30 months	M	ND	+	+	+	+	+	+	-	Roh et al. (2020)
		Old	27–28 months	F	ND	+	+	+	ND	ND	+	-	Dai et al. (2014); Campbell et al. (2019)
Hypertension	Dahl salt-sensitive (SS) rat	Young	19 weeks	M	ND	+	+	+	+	+	−	-	Doi et al. (2000)
		Young	26 weeks	M	ND	-	+	+	+	+	−	-	Doi et al. (2000)
	DOCA-salt–induced rat and mouse	Young	10–12 weeks	M	ND	+	+	+	+	−	+	-	Matsumura et al. (2000); Jeong et al. (2013); Basting and Lazartigues (2017); Bowen et al. (2017)
	Spontaneously hypertensive rat (SHR)	Old	18–24 months	M	ND	+/−*	+	+	+	+	ND	-	Pfeffer et al. (1979); Bing et al. (1995); Kuoppala et al. (2003); Kokubo et al. (2005)
	Angiotensin II infusion mouse	Young	12–13 weeks	M	ND	+	+	+	+	ND	+	-	Becher et al. (2012); Inoue et al. (2012); Westermann et al. (2012); Regan et al. (2015)
Metabolic stress (obesity and diabetes)	Ob/ob mouse	Young	10–11 weeks	F/M	ND	+	+	+	+	−	−	-	Christoffersen et al. (2003); Van den Bergh et al. (2008); Manolescu et al. (2014)
	Zucker rat	Young	14 weeks	F/M	+	+	+	+/−	+/−	−	ND	Fibrosis: absent in females; Hypertrophy: absent in males	Ren et al. (2000); Wang et al. (2005); van den Brom et al. (2010); Lum-Naihe et al. (2017)
	db/db mouse	Young	20–28 weeks	F/M	+	+	+	+	+	+	+	an increase in LV mass is enhanced in females, males exhibited microvascular rarefaction	Broderick et al. (2012); Ostler et al. (2014); Papinska et al. (2016); Alex et al. (2018)
Multi-hit models	Obese ZSF1 rat	Young	6–32 weeks	F/M	+	+	+	+	+	ND	+	more severe obesity, increased blood pressure in females	Nguyen et al. (2020); Schauer et al. (2020)
	HFD + l-NAME	Young	7 months	M(F)	+	+	+	+	+	+	+	Attenuated in females	Schiattarella et al. (2019); Tong et al. (2019); Schiattarella et al. (2021); Tong et al. (2021)
	HFD + ANG2	Old	21–24 months	F(M)	+	+/−	+	+	+	+	+	HFrEF in males	Withaar et al. (2021a)
	HFD + DOCP	Middle-age	16 months	M/F	ND	+	+	+	+	+	+	-	Deng et al. (2021)
Large animal models	Aortic-constriction in cats	Young	6 months	M	ND	+	+	+	+	+	ND	-	Wallner et al. (2017); Wallner et al. (2020)
	STZ + HFD + renal artery embolization in swine	Young	8–9 months	F	ND	+	+	ND	+	ND	ND	-	Sorop et al. (2018)
	Western diet + aortic banding in Ossabaw swine	Young	12 months	F	ND	+	+	+	+/−	+/−	ND	-	Olver et al. (2019)
	Western diet + DOCA in Gottingen Miniswine	Young	19 months	F	ND	+	+	+	+	+	ND	-	Sharp et al. (2021)

Abbreviations: STZ, streptozotocin; HFD, high-fat diet; l-NAME, N-nitro-l-arginine methyl ester; ANG2, angiotensin 2; DOCP, deoxycorticosterone pivalate; DOCA, 11-deoxycorticosterone acetate; F, female; M, male; ND, not determined. * Reduced ejection fraction in later stage. Methods used to assess each phenotype: preserved EF, diastolic dysfunction–transthoracic echocardiography, cardiac magnetic resonance imaging or pressure–volume (P-V) catheter technique, cardiac hypertrophy–echocardiography, heart weight measurement, gene expression of hypertrophy markers, cardiac fibrosis–histological staining and gene expression of fibrosis marker, lung congestion measurements of wet lung weight or lung wet/dry ratio, and exercise intolerance–treadmill running.

## HFpEF Animal Models for Preclinical Studies

Animal models of HFpEF should mimic the phenotypes and symptoms observed in patients with HFpEF. Diastolic dysfunction as well as concentric hypertrophy and fibrosis are the main cardiac characteristics for HFpEF. Patients with HFpEF also develop systemic derangements such as exercise intolerance and pulmonary congestion/edema. Different strategies for induction of HFpEF risk factors, such as aging, hypertension, and metabolic stress, have been used alone or in combination to reproduce diastolic dysfunction and other phenotypes of HFpEF in animal models.

## Single-Stressor Preclinical Models

### Aging

The aging heart shows phenotypic changes such as cardiac hypertrophy, diastolic dysfunction, worsened myocardial performance, and preserved systolic function at rest but impaired while performing exercise (Lakatta and Levy, 2003). The overlapping characteristics of cardiac aging and HFpEF and the sharp age-related increase in HFpEF prevalence support the importance of aging-based HFpEF models and the cross-talk between cardiac aging and HFpEF pathogenesis (Conceição et al., 2016; Beale et al., 2018).

Consistent with the observations in humans, studies on C57BL/6 mice (male, female, or mixed sex) showed that aging results in left ventricular and left atrial hypertrophy, diastolic dysfunction, worsened myocardial performance, and a modest decline in systolic function (Dai et al., 2009; Dai et al., 2014; Roh et al., 2020). In addition, old (>24 months) male or female C57BL/6 mice display exercise intolerance (Campbell et al., 2019; Chiao et al., 2020; Roh et al., 2020). Old or senescent C57BL/6 mice (>24 months) have a low incidence of conventional cardiovascular risk factors associated with HFpEF, e.g., they do not develop hypertension (Chiao et al., 2012; Roh et al., 2020), suggesting that some features of HFpEF develop with aging independent of these risk factors. Interestingly, these age-related changes can be reversed by caloric restriction, rapamycin treatment, and exercise training (Dai et al., 2014; Chiao et al., 2016; Roh et al., 2020).

Senescence-accelerated mouse (SAM) is a model of accelerated aging. Untreated male senescence-prone (SAMP8) mice develop diastolic dysfunction at 6 months of age (Reed et al., 2011), whereas untreated female SAMP8 mice display similar diastolic function as control senescence-accelerated resistant (SAMP1) mice (Gevaert et al., 2017). Female SAMP8 mice develop multiple HFpEF features on a high-fat and high-salt diet; however, the effects of the high-fat and high-salt diet in male SAMP8 mice and the sex-dependent differences in diastolic function at baseline were not determined (Gevaert et al., 2017).

Although aging animal models and patients with HFpEF share some common features such as cardiac hypertrophy, diastolic dysfunction, and exercise intolerance, aging alone does not result in HF in most animal models (Dai et al., 2009; Dai et al., 2014; Roh et al., 2020). However, as aging is a main risk factor of HFpEF, advanced age should be incorporated as an additional stressor in preclinical models of HFpEF to better mimic the patient population with HFpEF.

### Hypertension

Hypertension is one of the major comorbidities contributing to the development of HFpEF. Because of its structural and functional consequences on the cardiovascular system, hypertensive stress is widely used in different animal models to study HFpEF.

Dahl salt-sensitive (SS) rat model is a commonly used HFpEF model (Riehle and Bauersachs, 2019). Treatment of male Dahl SS rats with 8% NaCl high-salt diet starting 7–8 weeks of age elevated blood pressure and resulted in progressive LV hypertrophy, diastolic dysfunction, and preserved systolic function at 19 weeks of age (Doi et al., 2000; Klotz et al., 2006). However, when the diet treatment was continued to 26–27 weeks of age, the animals display reduced LVEF (Doi et al., 2000; Klotz et al., 2006). The progression of HFpEF into HFrEF is very uncommon in humans, and that limits the relevance of Dahl SS rats as a model for human HFpEF (Doi et al., 2000; Valero-Muñoz et al., 2017).

Deoxycorticosterone acetate (DOCA)–salt hypertension leads to hypervolemia due to an impairment of renal sodium handling where the kidneys reabsorb more water and sodium (Basting and Lazartigues, 2017). When young Wistar male rats were treated with DOCA and 1% sodium chloride water for 28 days, they develop hypertension, cardiac hypertrophy, and fibrosis (Matsumura et al., 2000; Basting and Lazartigues, 2017). Importantly, DOCA-salt–induced hypertension results in diastolic dysfunction and preserved systolic function in both rats and mice (Lovelock et al., 2012; Jeong et al., 2013; Bowen et al., 2017). Exercise intolerance has been reported in DOCA-treated mice but pulmonary congestion was not observed (Bowen et al., 2017).

Spontaneously hypertensive rat (SHR) is an inbred rat strain that has a predisposition to the spontaneous development of hypertension (Dickhout and Lee, 1998). At around 9 months of age, male SHR develops HFpEF features including diastolic dysfunction, cardiac hypertrophy, impaired myocardial performance, altered hemodynamic function, and pulmonary hypertension (Pfeffer et al., 1979; Bing et al., 1995; Kuoppala et al., 2003; Kokubo et al., 2005; Regitz-Zagrosek and Kararigas, 2017). However, as in the Dahl SS rat model, SHR transitions from HFpEF to HFrEF occur at 18–24 months of age, which is major limitation in the application of this model in HFpEF studies (Valero-Muñoz et al., 2017).

Angiotensin II plays a major role in cardiac homeostasis and regulates such processes as afterload, preload, cardiac hypertrophy, and fibrosis (Regan et al., 2015). Young male mice infused with angiotensin II develop hypertension and display preserved systolic function, cardiac fibrosis, increased LV hypertrophy, and diastolic dysfunction (Becher et al., 2012; Inoue et al., 2012; Westermann et al., 2012; Regan et al., 2015), which all are features of human HFpEF. Angiotensin II infusion can result in LV dilatation in Balb/c mice (Peng et al., 2011) or concentric hypertrophy in C57BL/6J mice, which better reflects HFpEF features observed in humans (Inoue et al., 2012; Westermann et al., 2012).

While animal models of hypertension display cardiac features of HFpEF at an early stage, extracardiac features of HFpEF are not well represented and some hypertensive models progress to HFrEF at a later stage (Pfeffer et al., 1979; Doi et al., 2000; Kuoppala et al., 2003; Kokubo et al., 2005; Klotz et al., 2006). Therefore, they are not considered as good models for HFpEF studies. In addition, most hypertensive models were investigated only in males, and sex differences of these models have not been determined.

### Metabolic Stress–Obesity and Diabetes

Obesity and diabetes are two of the major comorbidities in HFpEF (Altara et al., 2017). Patients with obesity or diabetes are more resistant to insulin and exhibit increased inflammation and dyslipidemia. All of the above conditions are strongly associated with the HFpEF (Altara et al., 2017).

Zucker rats (males or females depending on study) develop insulin resistance and dysfunctional leptin receptors due to a mutation in *Lepr* gene, which encodes the leptin receptor and that leads to the worsen affinity of leptin to its receptor (Kurtz et al., 1989). This mutation causes hyperphagia and obesity, but Zucker rats do not become diabetic. Zucker rats display hypertension, preserved LVEF, early diastolic dysfunction, cardiomyocyte hypertrophy, and fibrosis (Ren et al., 2000; Wang et al., 2005; van den Brom et al., 2010; Lum-Naihe et al., 2017). However, other HFpEF characteristics like lung congestion or exercise intolerance do not occur or have not been determined (van den Brom et al., 2010).

Ob/ob mouse is a leptin-deficient model in C57BL/6J background that spontaneously develops obesity and type 2 diabetes. Concentric hypertrophy, diastolic dysfunction, and preserved LVEF have been observed in ob/ob female and male mice (Christoffersen et al., 2003; Van den Bergh et al., 2008; Withaar et al., 2021b). However, ob/ob mice display normal exercise tolerance and expression of natriuretic peptides is unchanged or even slightly reduced compared to controls (Broderick et al., 2014; Manolescu et al., 2014; Withaar et al., 2021b).

Db/db leptin receptor-deficient mouse model is characterized by a point mutation in the gene that encodes the leptin receptor in C57BL/6J or C57BL/6KsJ background. This mutation leads to obesity and results in type 2 diabetes (Chen et al., 1996). At 6 months of age, db/db mice exhibit cardiac hypertrophy, fibrosis, diastolic dysfunction with preserved systolic function (Barouch et al., 2003; Alex et al., 2018). These HFpEF features are observed in both sexes but female db/db mice have exacerbated LV remodeling and hypertrophy compared the males (Alex et al., 2018). Exercise intolerance and pulmonary congestion are also observed in this model (Broderick et al., 2012; Ostler et al., 2014).

These models of metabolic stress have marked obesity with (ob/ob and db/db mice) or without diabetes (Zucker rats) and develop cardiac features of HFpEF in both sexes. Sex differences in cardiac HFpEF features have been observed, but age differences have not been determined in these models.

## Limitations of Single-Hit HFpEF Models

Early attempts on the development of HFpEF animal models mainly focused on the use of single perturbations (such as aging, hypertension, and diabetes/metabolic stress) to induce the clinical features of HFpEF. However, because HFpEF is a systemic disorder with multiple comorbidities, these single-hit models typically do not mimic certain symptoms of patients with HFpEF. Very recently, researchers established several novel models that utilize perturbations on two or more risk factors of HFpEF to recapitulate major clinical features of this complex syndrome (Table 1).

## Two- and Multi-Hit Models

Obese ZSF1 rat is one of the first models that combine more than one perturbation to induce HFpEF phenotype in rodents. This model established by crossing female Zucker diabetic rat and male spontaneously hypertensive HF rat develops both cardiac and extracardiac HFpEF phenotype (Hamdani et al., 2013; Nguyen et al., 2020; Schauer et al., 2020). Nguyen et al. showed that both male and female obese ZSF1 rat developed HFpEF phenotypes features such as diastolic dysfunction, cardiac hypertrophy, and fibrosis (Nguyen et al., 2020).

Recently, Schiattarella et al. (2019) induced concomitant metabolic and hypertensive stress (two-hit stress) in male C57BL/6N mice with high-fat diet and inhibition of constitutive nitric oxide synthase using N-nitro-l-arginine methyl ester (l-NAME), respectively. After 15-week treatment, two-hit stress–treated mice display preserved EF and significant diastolic dysfunction accompanied by extracardiac HFpEF phenotypes including pulmonary congestion and exercise intolerance (Schiattarella et al., 2019). This model also displays cardiac and cardiomyocytes hypertrophy, fibrosis, and reduction in myocardial capillary density (Schiattarella et al., 2019). Compared to l-NAME-only– and HFD-only–treated mice, two-hit stress–treated mice exhibit more severe HFpEF phenotypes and developed impaired cardiomyocyte contraction and relaxation kinetics (Schiattarella et al., 2019). Using this model, the authors showed a critical role of nitrosative stress in the pathophysiology of HFpEF (Schiattarella et al., 2019).

L-NAME and HFD on female mice resulted in significantly attenuated phenotype as compared to male counterparts (Tong et al., 2019). Female mice treated with two-hit stress mice showed modest decline in diastolic function, mild fibrosis, and maintained normal cardiac mass and LV remodeling index. Exercise intolerance, but not lung congestion, was observed in two-hit stress treated females (Tong et al., 2019). Milder HFpEF phenotypes reported in females are consistent with observations from many conditions such as myocardial ischemia and pressure or volume overload, where the female sex is associated with better cardiovascular disease outcomes (Regitz-Zagrosek and Kararigas, 2017). The protective effect of the female sex in this HFpEF model is in contrast to the higher or equal risk of HFpEF in women (Ho et al., 2016; Dunlay et al., 2017). Importantly, ovariectomized two-hit stress–treated females did not show any differences in HFpEF phenotypes, suggesting that estrogens do not mediate the protection of the female sex in this model (Tong et al., 2019). Age is a major risk factor of HFpEF, but this two-hit stress model has only been performed on 8-week-old mice. It is very likely that aged animals treated with l-NAME and HFD would present exacerbated HFpEF progression, including the development of significant HFpEF phenotypes in old females. This two-hit model was later used to demonstrate the roles of NAD metabolism and Xbp1s-FoxO1–regulated lipid accumulation in the pathogenesis of HFpEF (Schiattarella et al., 2021; Tong et al., 2021).

A recent study by Withaar et al. (Withaar et al., 2021a) combined advanced age (18–22 months old), cardiometabolic stress (12-week HFD treatment), and hypertensive stress (4-week angiotensin 2 infusion) to induce HFpEF development in female mice. This model is characterized by preserved LVEF, concentric LV hypertrophy, increased LV fibrosis, reduced diastolic function, atrial enlargement, pulmonary congestion, and elevated natriuretic peptides, which all are characteristic for patients with HFpEF (Withaar et al., 2021a). The authors used this preclinical model to show that selected metabolic drugs can normalize the cardiometabolic HFpEF phenotypes (Withaar et al., 2021a). Interestingly, using this protocol on male mice resulted in eccentric remodeling, cardiac dilatation, and reduced systolic function, all characteristics of HFrEF (Withaar et al., 2021a). Thus, future studies are needed to reveal factors contributing to the sex differences and the mechanisms underlying the higher susceptibility of females to HFpEF phenotype and males to HFrEF.

A more recent study by Deng et al. (2021) developed a similar model where 3-month-old mice were fed with HFD for 13 months and injected a bolus of desoxycorticosterone pivalate (DOCP) in the last month to accentuate hypertension and systemic inflammation. At the end of treatment, mice were characterized by high blood pressure as well as increased oxidative stress and inflammation (Deng et al., 2021). Peripheral abnormalities such as exercise intolerance and pulmonary congestion were also observed (Deng et al., 2021). Heart muscle displayed significant hypertrophy, fibrosis, and diastolic dysfunction with unaffected LVEF (Deng et al., 2021). These changes resemble the typical hemodynamic and peripheral features of patients with HFpEF. Using this model, the authors showed that the interplay between mitochondrial protein hyperacetylation and inflammation is a pathogenic mechanism of HFpEF (Deng et al., 2021). The study was performed on mixed sex mice, and potential sex differences were not investigated (Deng et al., 2021).

The aforementioned two- and multi-hit strategies generate novel animal models that display typical cardiac and extracardiac features of human HFpEF patients. Sex differences in HFpEF phenotypes were observed in two of these models (Tong et al., 2019; Withaar et al., 2021a), and advanced age was incorporated as a stressor in the two multi-hit models (Withaar et al., 2021a; Deng et al., 2021). Further investigations on age and sex differences in these models will offer critical insights into how age and sex impact HFpEF pathogenesis.

## Large Animal Models of HFpEF

The aforementioned murine and rat models have some limitations due to their sizes and different cardiac structure and function as compared to large animals and humans (Riehle and Bauersachs, 2019). Large animal preclinical models, including feline, canine, and swine, have been used to investigate the pathogenic mechanisms of HFpEF (Table 1). Early large animal models of HFpEF were based on single-hit perturbations, such as pressure overload in sheep (Charles et al., 1996) or canine model of mild coronary microembolizations (He et al., 2004), and exhibit only a portion of HFpEF features. Recently, slow-progressive pressure overload by aortic-constriction was used to establish a feline HFpEF model (Wallner et al., 2017; Wallner et al., 2020; Gibb et al., 2021). Comprehensive phenotyping of this model showed many features of HFpEF, such as concentric LV hypertrophy and significant diastolic dysfunction (Wallner et al., 2017; Wallner et al., 2020). These cardiac phenotypes are associated with LV fibrosis, cardiomyocyte hypertrophy, and elevated NT-proBNP plasma levels (Wallner et al., 2017; Wallner et al., 2020). Moreover, this model develops signs of pulmonary hypertension and vascular remodeling (Wallner et al., 2017). Using this model, Wallner et al. demonstrated the protective effects of histone deacetylase inhibition on cardiopulmonary structure, function, and metabolism (Wallner et al., 2020).

In recent years, multi-hit swine HFpEF models were also established. Using streptozotocin injection, HFD and renal artery embolization, Sorop et al. (2018) induced diabetes, hypercholesterolemia, and hypertension in female swine. Chronic exposure to these HFpEF comorbidities results in the development of cardiac fibrosis, cardiomyocytes stiffness, and diastolic dysfunction without changes in LVEF (Sorop et al., 2018). Systemically, animals present dyslipidemia, renal dysfunction, and systemic inflammation (Sorop et al., 2018). Similar phenotypes are observed in another swine model (Olver et al., 2019), where cardio-metabolic HF is induced in Ossabaw swine by aortic banding and Western diet (WD) feeding. Very recently, a noninvasive HFpEF model was developed by Sharp et al. (2021) using the combination of WD-induced obesity and DOCA-salt–induced hypertension in female Gottingen minipigs (known for its susceptibilities toward obesity, metabolic syndrome, and atherosclerosis). Severe LV diastolic dysfunction, concentric hypertrophy, and myocardial fibrosis are observed, whereas LVEF is preserved. In addition to these cardiac phenotypes, pulmonary and systemic hypertension and derangements in multiple organs, including pancreas, liver, and kidneys, are observed (Sharp et al., 2021).

Despite sharing more similarities in their cardiovascular physiology with humans, large animal preclinical models have their limitations. Primary limitation of large animal preclinical models is the high general cost compared to rodent models (Riehle and Bauersachs, 2019). This often leads to lower number of animals used in the studies. In each of studies described above, either male or female sex was investigated, and potential sex differences were not determined. Another limitation is the lack of established protocols to measure some endpoints such as exercise intolerance, which was also not investigated in aforementioned studies. Finally, aging in large animals takes longer compared to rodents, so incorporation of aging as a risk factor and determination of age-dependent mechanisms are more time-consuming and costly. In all the abovementioned large animal studies, only young/adult animals were analyzed.

## Discussion

Most of the pharmacological and device therapies for HFpEF investigated in completed clinical trials have been shown to be ineffective. So far, only the recent EMPEROR-Preserved clinical trial showed that empagliflozin reduced the combined risk of cardiovascular death or hospitalization for HF in patients with HFpEF (Anker et al., 2021). It is important to note that some studies suggested effectiveness of certain treatments in specific subgroups of patients with HFpEF (Lam et al., 2012; Solomon et al., 2016; Solomon et al., 2019). For example, the PARAGON-HF trial showed that sacubitril–valsartan treatment did not reduce the rate of total hospitalizations for HF and death from cardiovascular causes among patients with HF with a LVEF of 45% or higher (Solomon et al., 2019). However, when trial outcomes were analyzed among different subgroups of patients, a potential benefit was suggested in patients with a LVEF of 45%–57% and in women.

It is highly unlikely that a single animal model will fully recapitulate the phenotypes of the heterogenous HFpEF population. For example, a limitation of HFpEF models induced by hypertension is the fact that, in the majority of patients with HFpEF, it continues to display HFpEF symptoms even when their blood pressure is under control (Conceição et al., 2016). Some models transition from HFpEF to HFrEF, which is a rare phenomenon in humans and limits their clinical relevance (Valero-Muñoz et al., 2017; Mishra and Kass, 2021). Although some of described models are very complex and include multiple comorbidities like aging, obesity, and hypertension, they are unable to represent the whole spectrum of patients with HFpEF, who can present different combinations of these comorbidities and others such as COPD, atrial fibrillation, and renal dysfunction (Dunlay et al., 2017). The differential treatment effects in different patient subgroups in clinical trials and the limitations of various preclinical HFpEF models highlight the need to develop animal models to represent specific HFpEF subphenotypes. Therefore, rather than looking for a universal HFpEF model, future studies should utilize various animal models to study the pathogenic mechanisms and develop therapeutics for different human HFpEF subphenotypes.

Many preclinical HFpEF studies used animals of one sex or mixed sex, limiting the interpretation of sex-specific differences in HFpEF pathogenic mechanisms. Because of the sex disparities in HFpEF, it is important to include both sexes in HFpEF research and analyze the outcomes separately to determine any potential sex-specific mechanisms. While it is well established that the prevalence of HFpEF sharply increases with age (Owan et al., 2006; Oktay et al., 2013; Dunlay et al., 2017), the majority of the preclinical HFpEF studies used young animals. Although it is expensive and time-consuming to age the animals to the appropriate ages, it is important to include this risk factor in preclinical studies. In fact, the need for improved animal models, which incorporate the effects of aging and its associated comorbid conditions, has been recently postulated as one of the research priorities for HFpEF by a National Heart, Lung, and Blood Institute Working Group (Shah et al., 2020). Incorporating aging in preclinical HFpEF models will provide crucial insights into the age-related derangements that predispose the elderly to HFpEF pathogenesis. This information will be invaluable to the development of new therapies for HFpEF in older adults, who are most at risk for the disease.
